# Supplementary material for: The Observation of Ligand-Binding-Relevant Open States of Fatty Acid Binding Protein by Molecular Dynamics Simulations and a Markov State Model
Source: Int J Mol Sci. 2019 Jul 15;20(14):3476. doi: 10.3390/ijms20143476 (PMC6678811; doi:10.3390/ijms20143476)
Supplement: Supplementary file 1 [file ijms-20-03476-s001.pdf]

Supplementary materials

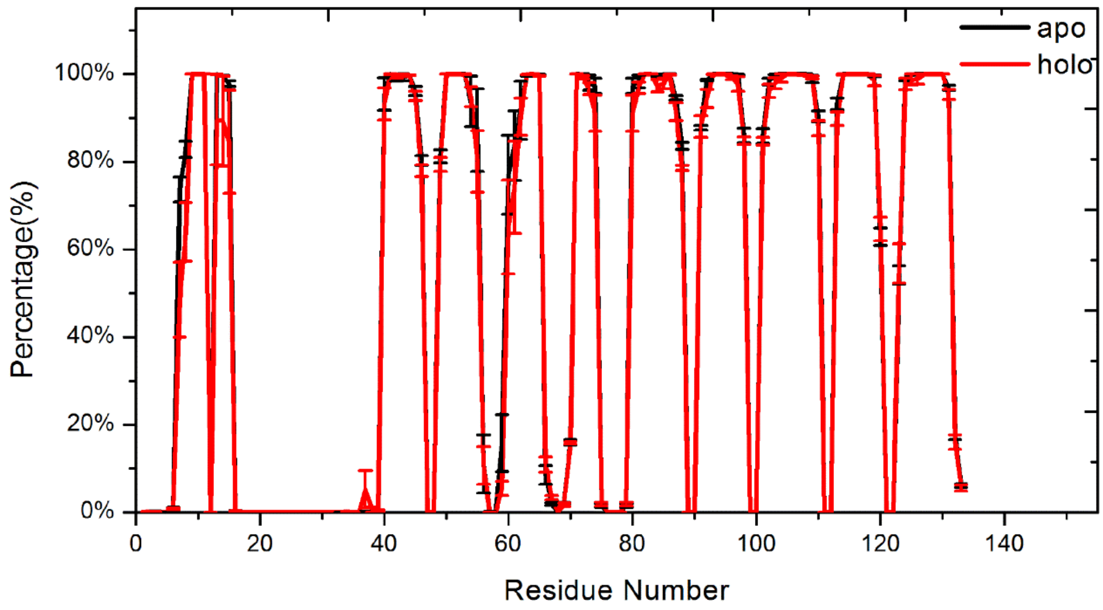

Figure S1. The β-strand propensity of residue on HFABP.

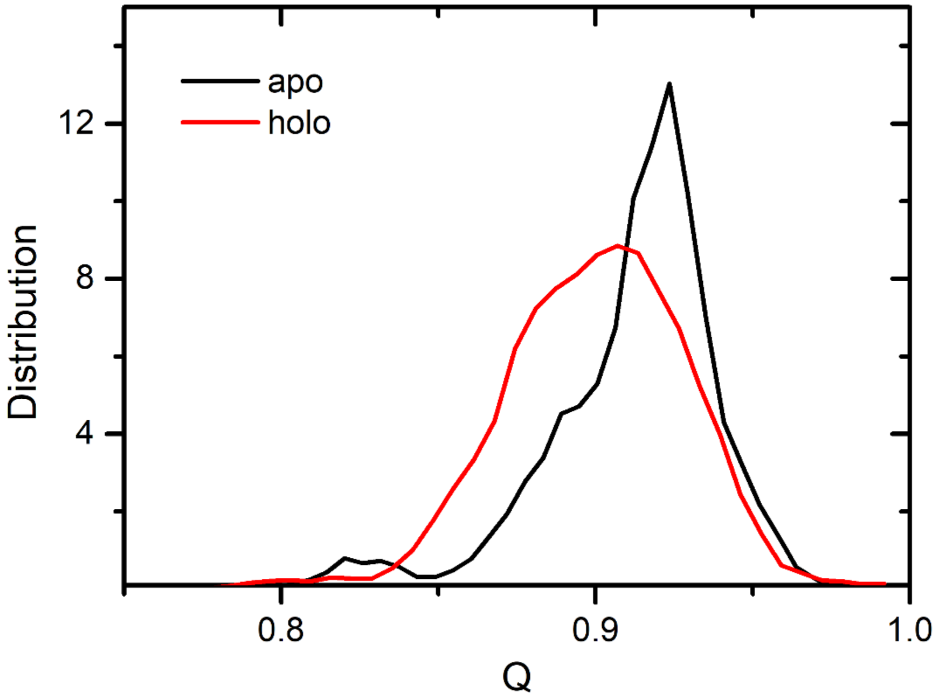

Figure S2. The distributions of fraction of native contact (Q).

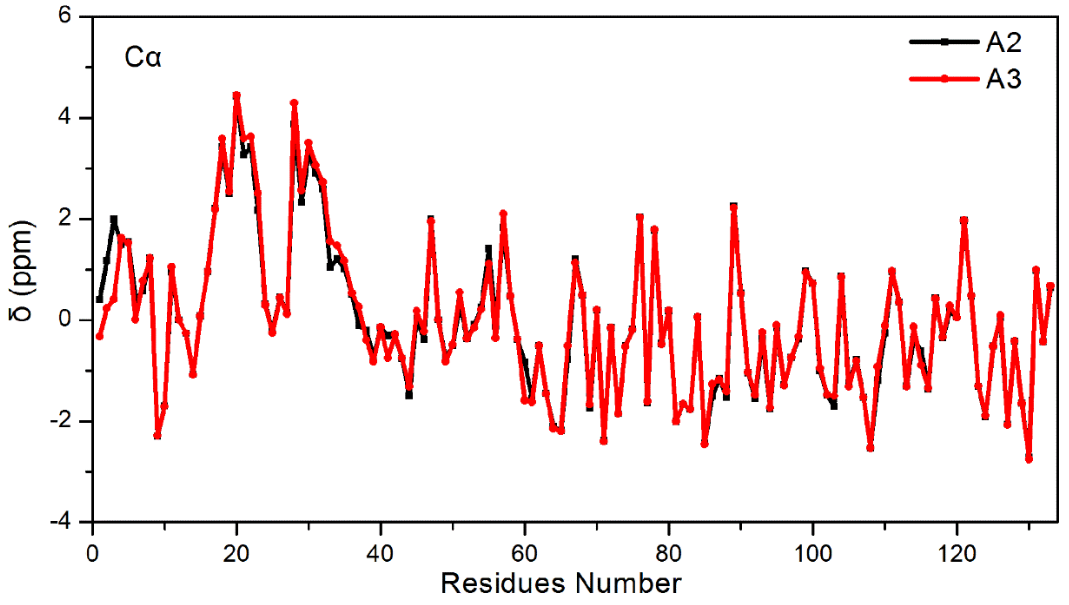

Figure S3. The Ca-atom secondary chemical shift of the conformations in the state A2 and A3 of apo-HFABP.

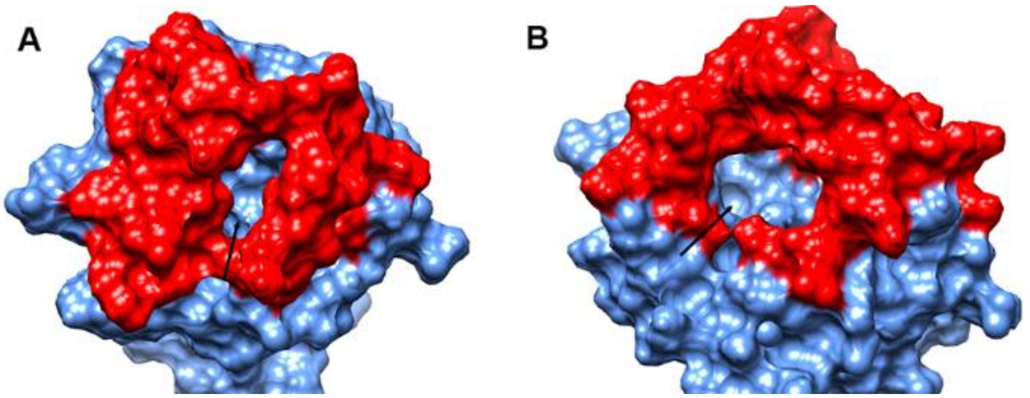

Figure S4. The surface representation of "open" intermediates. (A) Example of intermediate structure of apo-HFABP; (B) Example of intermediate structure of holo-HFABP. The portal regions were colored in red, and the open gates were point by the black arrows.

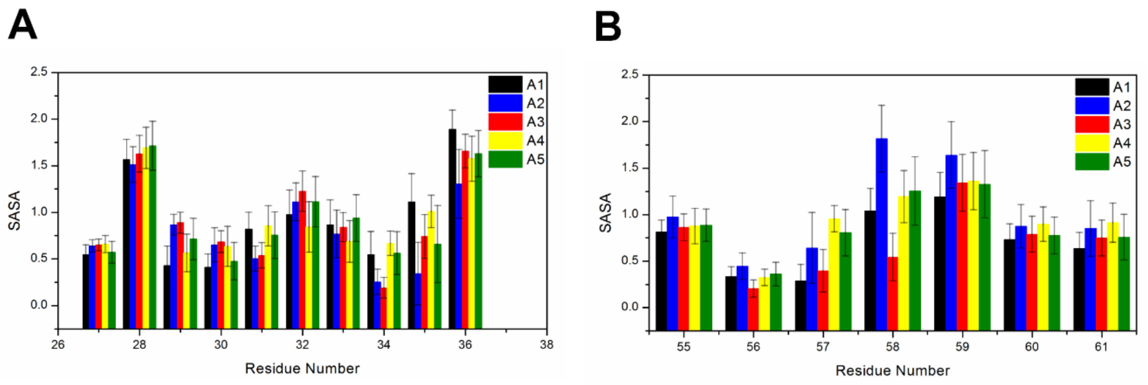

Figure S5. The residue solvent accessible surface area (SASA) in different states. (A) SASA values of residues on the α2 helix region. (B) SASA values of residues on the βC-βD loop region.

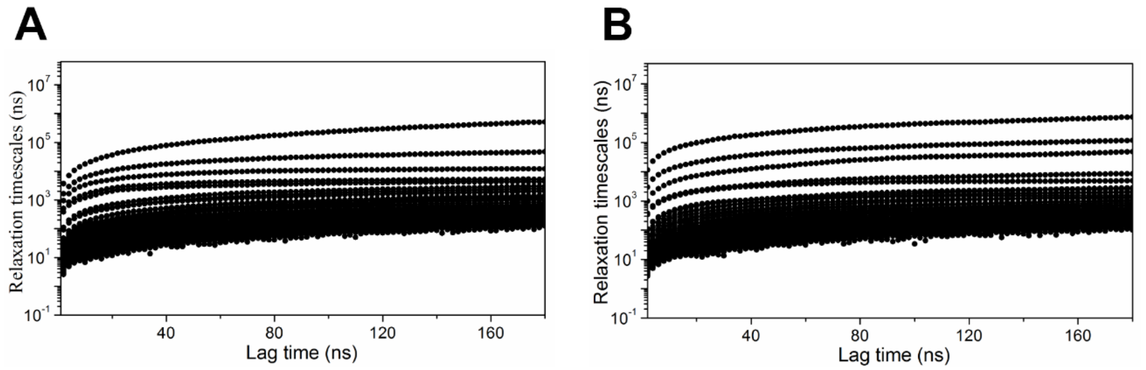

Figure S6. Relaxation time scale as a function of lag time of the microstates. (A) apo-HFABP; (B) holo-HFABP.

Table 1. The Cα RMSD of residue 15-120 between intermediate states (unit: Angstrom).

	H1	H2	H3	H4	H5	H6
A1	2.2	3.73	1.15	3.58	2.64	4.22
A2	1.74	3.95	2.60	2.97	2.52	3.63
A3	1.41	3.40	2.23	2.83	1.86	3.33
A4	1.70	3.51	1.94	3.13	1.98	3.72
A5	2.76	3.29	1.99	3.67	2.9	4.00

Table S2. The clustering analysis of all conformations from apo- and holo-HFABP simulations

	**A1[b]**	**A2**	**A3**	**A4**	**A5**	**H1[c]**	**H2**	**H3**	**H4**	**H5**	**H6**
**C1[a]**	20.0%	7.0%	5.5%	8.0%	8.0%	9.0%	8.0%	28.0%	2.5%	4.0%	0
**C2**	0.2%	1.5%	9.0%	3.0%	2.0%	17.0%	27.0%	27.0%	2.5%	10.0%	0
**C3**	0	0	0	0	0	0	0	0.2%	66.0%	0.2%	34%
**C4**	3.0%	9.0%	58.0%	0.3%	2.5%	0.3%	0.6%	1.0%	25.0%	0	0
**C5**	0	0	0	0	0	0	0	99.0%	0.6%	0	0

[a]. The largest five clusters, i.e. C1 to C5;

[b]. The intermediates of apo-HFABP obtained by MSM analysis;

[c]. The intermediates of holo-HFABP obtained by MSM analysis;

[d]. The elements in the table corresponding to the percentages of conformations in the clustering (C1 to C5) from the MSM intermediates.
